# Homocysteine-induced macrophage inflammatory protein-2 production by glomerular mesangial cells is mediated by PI3 Kinase and p38 MAPK

**DOI:** 10.1186/1476-9255-6-27

**Published:** 2009-09-26

**Authors:** Suresh Shastry, Leighton R James

**Affiliations:** 1 Department of Medicine, University of Texas Southwestern Center, Dallas, TX, USA

## Abstract

**Background:**

Homocysteine (Hcy) and inflammatory cytokines have been linked to adverse outcomes in persons with cardiovascular and kidney diseases and recent reports suggest that cytokine-mediated inflammatory infiltrates may be an important contributor to the pathogenesis the aforementioned diseases. Although some reports suggest that Hcy directly influences inflammatory cytokine production, this proposition has not been supported by data from other studies. The objective of the current study was to a) utilize an *in vitro *cellular model to identify cytokines that may be affected by Hcy and b) examine the role of mitogen activated protein kinase (MAPK) and phosphatidyl inositol 3- (PI3) Kinase in Hcy modulated cytokine production.

**Methods:**

Primary rat glomerular mesangial cells (MC, passage 8 to 15), isolated by standard sieving methodology, were exposed to Hcy (15, 50 or 100 μM) with L-cysteine (L-Cys; 100 μM) serving as a control. An antibody array was used to identify cytokines that were modulated when MCs were exposed to Hcy. Gene expression was assessed by quantitative RT-PCR, while western blotting analysis was used to assess cellular protein levels in the presence and absence of inhibitors of MAPK and PI3 Kinase. Finally, leukocyte adhesion assay was used to examine the effect of Hcy on leukocyte adhesion to glomerular MCs that were maintained in media without, and with, kinase inhibitors.

**Results:**

We identified macrophage inflammatory protein 2 (MIP-2) as a key cytokine that manifested increases in both protein and mRNA following exposure of glomerular MC to pathophysiologic Hcy levels (50 μM). Further analyses revealed that Hcy-induced MIP-2 was dependent on activation of p38 MAPK and PI3 kinase. MIP-2 enhanced leukocyte adhesion to MC and this MIP-2-enhanced leukocyte adhesion was also dependent on activation of p38 MAPK and PI3K. Finally, we demonstrate that leukocyte adhesion to MC is specifically inhibited by anit-MIP2 antibody.

**Conclusion:**

The data suggest that Hcy participates in inflammatory cytokines production by glomerular MC and that Hcy-induced MIP-2 mediates leukocyte adhesion to MC.

## Background

Elevated levels of plasma homocysteine (Hcy; ≥15 μM) are associated with chronic kidney disease and end-stage renal disease (ESRD) irrespective of the underlying aetiology [1,2]. However, the pathophysiological consequences of hyperhomocysteinemia (Hhcy) remain controversial because, although Hhcy has consistently been associated with morbidity and mortality, recent epidemiologic studies have produced conflicting results. In a prospective community-based study of persons without kidney disease at study inception, over a 5-year period, chronic kidney disease risk was found to increase in association with escalating Hcy levels in both men and women [3]. The converse has been also reported; that is, chronic kidney disease is a direct cause of Hhcy; Hcy levels rises in direct relationship to reduction in glomerular filtration rates (GFR) [4,5]. Given the existence of these inconsistent observations, the role of Hcy in progressive kidney disease is unresolved and continues to be the focus of ongoing clinical and basic investigations.

Notwithstanding contradictory observations, studies have identified an association between Hcy and inflammation. For instance, in subject aged ≥ 65 years, IL-6 and IL-1ra cytokines were independent predictors of plasmatic Hcy concentrations [6]. Similarly, in another study, serum Hcy levels and C-reactive protein levels were significantly higher in patients with stage 3 chronic kidney disease (CKD) compared to those with stage 1 disorder [7]. In this regard, the potential consequences of Hhcy on inflammation in the kidney have been studied by assessing the impact of Hcy on monocyte chemoattractant protein-1 (MCP-1) expression by glomerular mesangial cells (MC) [8]. Hcy (50 to 200 μM) induced MCP-1 protein and mRNA levels in glomerular MC via nuclear factor kappa B (NF-κB) activation, a process found to be mediated by generation of oxidative stress [8].

In a related study, the same investigators observed that in methionine-induced Hhcy rats, MCP-1 protein and mRNA levels were increased in kidneys and that this increase was dependent on NF-κB. The authors surmised that these observations link Hcy-induced inflammatory response to kidney injury and progressive kidney disease.

We have demonstrated that Hcy induces DNA damage and apoptosis in MC. These adverse effects were dependent on Hcy-induced oxidative stress and p38 MAPK activation [9]. In addition, in a separate study, we have also documented calcium-dependent, extracellular signal-regulated kinase mediated MC proliferation in response to Hcy [10]. These prior studies suggest that elevated levels of Hcy may contribute to MC proliferation or apoptosis, processes that may mediate kidney injury and contribute to chronic kidney disease.

Given the observation that MC are able to secrete chemokines in response to extracellular stimuli, it has been proposed that these chemokines serve an important role of mediating leukocyte infiltration that participate in glomerular response to injury and in the progression of kidney disease [11]. Indeed, in circumstances where MC are exposed to noxious stimuli, they secrete macrophage inflammatory protein 2 (MIP-2, also known as CXCL2) that mediate neutrophil infiltration [12].

MIP-2 is a potent neutrophil chemotactic stimulant that is typically secreted by macrophages in response to inflammation induced by endotoxin [13]. MIP-2 is a member of the CXC chemokine sub-family of cytokines that includes IL-8 (CXCL8) and KC (CXCL1) among others. Structurally, CXC chemokines are characterised by possessing one amino acid residue between the first two conserved cysteine residues. This is in contrast to the CC chemokines (includes macrophage chemoattractant proteins [MCP] - 1, 2, 3, 4, regulated upon activation normal T cell expressed and secreted [RANTES], MIP-1α, β, γ, δ and MIP-3α and β) in which the first two conserved cysteine residues are adjacent [14,15]. The CXC chemokines are capable of regulating all stages of neutrophil recruitment (mobilization from bone marrow, tumbling and adhesion to the endothelium and transmigration) to inflammatory or injury foci; their actions are mediated by CXC receptors (CXCR) [16,17].

MCs are capable of producing and secreting MIP-2 and, MC-derived MIP-2 has been demonstrated to mediate glomerulonephritis in a rat model of the aforementioned disorder [12]. Accordingly, the current study had two major objectives namely a) to examine the role of Hhcy in cytokine production by MC and b) to define some of the signalling mechanism(s) that may participate in this processes. In particular, given our earlier observation that MC response to extracellular Hcy involves activation of MAPK, the role of MAPK activation in MIP-2 production by MC was evaluated.

## Methods

### Cell Culture

Sprague-Dawley rat MCs were isolated by the sieving method [18]. The cells were cultured in Dulbecco's Modified Eagle's Medium (DMEM) supplemented with 10% fetal bovine serum (FBS) (Invitrogen, CA), streptomycin (100 μg/ml), penicillin (100 IU/ml) and 2 mM glutamine at 37°C in 95% air/5% CO_2_. Cells from passage 8-15 were used throughout these studies. All other chemicals were obtained from Sigma-Aldrich (St. Louis, MO) unless otherwise indicated.

### Cytokine Antibody Array

A rat cytokine antibody array (Cat# R0608001; RayBiotech Inc., Norcross, GA, USA) was employed to assess cytokine production by MC following exposure to Hcy. The protocol was executed according to the manufacturer's specifications. Briefly, MCs (10^6 ^cells/100 mm dish) were initially seeded unto plastic dishes in DMEM supplemented with FBS (10%). Subsequently, cultures were serum-starved overnight (DMEM supplement with 0.5% FBS), followed by incubation in medium (DMEM supplement with 0.5% FBS) with L-cysteine (L-Cys; 100 μM) or Hcy (50 μM) for 24 hours at 37°C. The cells were harvested and cellular protein was prepared from lysates as described below. Protein form lysates (50 μg) was used to determine chemokine production using rat cytokine antibody array membranes according to the manufacturer's protocol. Membranes were initially blocked (30 minutes/room temperature), followed by exposure to cell lysate (2 hours/room temperature). After washing, exposure to biotin conjugated cytokine antibody and HRP-conjugated streptavidin, cytokines were detected using standard chemiluminescent methods (please see section below on 'Determination of MIP-2 protein'). The procedure was performed three times.

### Determination of MIP-2 expression by Mesangial Cells

MC were initially seeded unto plastic dishes (1 × 10^6 ^cells/100 mm dish) in DMEM supplemented with 10% FBS. Subsequently, cultures were serum-starved overnight, followed by incubation with L-cysteine (L-Cys; 100 μM) or Hcy (15 μM, 50 μM and 100 μM) for 24 hours at 37°C. Cells were harvested and total RNA was isolated by established methods [19]. Following cDNA synthesis (qPCR cDNA Synthesis Kit Cat# 600559, Stratagene, La Jolla, CA), qPCR was performed using an iQ-SYBR Green kit (Bio-Rad, Hercules, CA). MIP-2 expression was assessed using the following primers: *sense *- AACAAAC TGCACCC AGGAAG and *antisense *- GAGCTGGCCAATGCATATCT. GAPDH served as control; expression of the latter was determined using the following primers:- *sense *AGGTCGGTGTGAACGGATTTG and *antisense *- TGTAGACCATGTAGTTGAGGTCA. Gene expression was quantified by the standard curve method [20,21].

### Detection of MIP-2 Protein in Mesangial cells

Cultures were serum-starved overnight, followed by incubation with L-Cys (100 μM) or Hcy (15 μM, 50 μM and 100 μM) for 24 hours at 37°C. Subsequently, cells were washed with phosphate buffered saline (PBS; 4°C) and harvested under non-denaturing conditions by incubation (4°C/5 minutes) with lysis buffer (20 mM Tris, pH 7.4; 150 mM NaCl; 1 mM EDTA; 1 mM EGTA; 1%Triton X-100; 1 mM-glycerolphosphate, 1 mM Sodium Orthovanadate; 1 μg/ml leupeptin; 1 mM phenyl methylsulphonyl flouride). Following centrifugation (14,000 × g, 4°C, 10 minutes), the supernatant was transferred to a fresh microcentrifuge tube and the protein concentration was measured with Bio-Rad protein assay reagent (BioRad, Hercules, California, USA).

Protein was separated on a SDS-PAGE gel (4-20%). After electroblotting to a nitrocellulose membrane (Protran, Schleicher and Schuell, Keene, NH), membranes were incubated (room  temperature/3 hours) with 25 ml of blocking buffer (1× Tris buffered saline, TBS; 0.1% Tween-20 containing 5% w/v non-fat dry milk) and then overnight at 4°C with rabbit polyclonal macrophage inflammatory  protein-2 antibody (1:2000, cat #ab9777; Abcam, Cambridge MA) in 20 ml of antibody dilution buffer (1× TBS, 0.1% Tween-20) with gentle rocking. Membranes were washed 3 times with TTBS and then incubated with HRP-conjugated anti-rabbit secondary antibody (1:10,000, Cell Signalling Technology) in 20 ml of antibody dilution buffer (1× TBS, 0.1% Tween-20/60 minutes/room temperature). After three further TBS washes, the membrane was incubated with ECL Chemiluminescence Reagent (Amersham Biosciences) and then exposed to X-ray film (X-OMAT, Kodak, Rochester NY). Immune complexes were removed from the membrane by treatment with stripping buffer (100 mM 2-mercaptoethanol, 2% SDS, 62.5 mM Tris-HCl [pH6.7]; 50°C; 30 minutes). Subsequently, protein loading was assessed by re-blotting with anti-actin antibody (1:12,000 Sigma-Aldrich, St. Louis, MO.) and an HRP-conjugated anti-rabbit secondary antibody (1:25,000; Cell Signalling Technology). Protein bands were quantified using BioRad Quantity One software package.

In order to study the effect of kinase inhibitors on MIP-2, MCs were incubated in the presence of Hcy (50 μM) with or without inhibitors U0126 (p42/44 MAPK inhibitor; 10 μM), SB203580 (p38MAPK inhibitor; 10 μM) and LY294002 (PI3 Kinase inhibitor; 10 μM) for 24 h at 37°C. Subsequently, cells were washed with PBS (4°C) and harvested under non-denaturing conditions by incubation (4°C/5 minutes) with lysis buffer as described above. MIP-2 protein was quantified after detection by western blot as described above.

### Immunofluorescence Microscopy for MIP-2

MCs (10^4 ^cells/well) were initially plated onto sterile two-chambered slides (product no. 154461, Nalge Nunc, Rochester, NY) exactly as described for other experiments above. After incubation (37°C; 24 hours) in the presence of Hcy (50 μM) with or without kinase inhibitors, cells were washed (thrice with 1× PBS) and fixed (3.7% formaldehyde, 10 minutes, ambient temperature). Following PBS washes (thrice), cells were permeabilized (0.1%Triton X-100, 4°C, for 2 minutes), washed again with PBS and incubated with blocking solution (1% BSA; 1% goat serum in PBS) for 60 minutes at room temperature.

The cells were subsequently incubated with rabbit polyclonal MIP-2 antibody (4°C; 24 hours) constituted in blocking solution. Following PBS washes (thrice), cells were incubated (60 minutes; ambient temperature; light-protection) with Alexa-fluor 555-conjugated goat anti-rabbit secondary antibody (Molecular Probes/Invitrogen). The cells were washed with PBS and slips were mounted onto glass slides using mount media anti-fade mixture and stored (4°C, light-protected) until fluorescence microscopy laser scanning was performed using a Zeiss Axioplan 2 Imaging System (Carl Zeiss MicroImaging Inc., Thornwood, NY, USA).

### Western Blot analysis of p38MAPK and p85 PI3K phosphorylation

Cultures were serum-starved overnight prior to the addition of L-Cys (100 μM) or Hcy (15 μM, 50 μM and 100 μM). Subsequently, cells were washed with PBS (4°C) and harvested under non-denaturing conditions by incubation (4°C/5 minutes) with lysis buffer as described above. Western blot was performed as described above. The immuno-blot membrane was incubated with anti-pp85 or anti-pp38 MAPK at 1:1000 dilution (overnight; 4°C), followed by incubating with HRP-conjugated anti-rabbit secondary antibody at 1:2000 for 60 minutes at room temperature. The membrane was reprobed with anti-p85 or anti-p38MAPK (dilution 1:1000), followed by incubating with HRP-conjugated anti-rabbit secondary antibody. The bands of pp85PI-3 K and pp38MAPK were normalized with p85 PI-3K and p38MAPK respectively for analysis using BioRad Quantity One package.

### Mouse Leukocyte adhesion assay

The assay was used to evaluate leukocyte-MC adhesion in the presence of increasing doses of Hcy, and Hcy (50 μM) with kinase inhibitors (SB203580 and LY294002) and pAb MIP-2. MCs were initially plated at a density of 10,000 cells/well in 24-well tissue culture plate. Following overnight serum starvation MCs were incubated (37°C; 24 hours) in the presence of Hcy (50 μM) with or without inhibitors 10 μM SB203580 (p38MAPK inhibitor) and 10 μM LY294002 (PI3 Kinase inhibitor).

Cell adhesion assay was performed as per manufacturer's protocol (Vybrant Cell Adhesion Assay Kit; Cell Biolabs Inc., San Diego, CA). In brief, leukocytes were isolated from blood collected from anaesthetized mice and prepared as described in the manufacturer's protocol (Easy lyse whole blood Erythrocyte Lysing Kit; Leinco Technologies Inc. St. Louis, MO). Subsequently, isolated leukocytes were labelled with Calcein AM, MCs were washed with PBS, followed by addition of labelled leukocyte cell suspension (13,000 cells/well) in DMEM to each well. The co-culture was incubated (2 hour, 37°C), and following this period, non-adherent cells leukocytes were removed by gently washing with PBS, followed by addition of 300 μl PBS to each well. Fluorescence from leukocytes bound to mesangial cells was determined by spectrophotometry (Wallac Victor, 1420 Multilabel counter, Perkin Elmer). The percentage of bound leukocytes to un-stimulated MC represented 100% and was compared to other conditions.

For neutralization experiments, MC stimulated with 50 μM Hcy overnight were washed with PBS. The cells were then incubated with 5 μg/ml pAb MIP-2 prepared in DMEM for 3 hours at 37°C, before incubating with labelled leukocytes.

### Statistical Analyses

In each series of experiment, differences between means were analyzed by Student's *t *test using Instat Statistical software (GraphPad Inc.San Diego, CA). Differences were considered significant at p < 0.05.

## Results

### Homocysteine influences cytokine levels in mesangial cells

Previous studies have suggested an association between Hcy and expression of inflammatory cytokines [12]. We sought to assess this relationship in the context of glomerular disease by utilising cytokine antibody array to register changes in cytokine levels. MC were exposed to pathophysiologic Hcy concentration (50 μM) that has been previously shown to modulate MC behaviour [10]. The results (table 1) revealed that several cytokines were significantly affected by this manoeuvre, including TIMP-1, MIP-2, interferon gamma and fractalkine. MIP-2 influences leukocyte migration and has been shown to mediate inflammatory infiltration in glomerular disease [22,23]. Accordingly, we chose to explore the influence of Hcy on MIP-2 and to relate the observations to leukocyte interaction with glomerular MC in an *in vitro *assay system.

**Table 1:** Antibody Array analysis of changes in cytokine levels in mesangial cells following exposure to DL-homocysteine (50 μM).

**Cytokine**	**Change**	**P-value**
TIMP-1	1.9	0.02
TNFα	1.1	NS
β-NGF	1.1	NS
MIP-3α	1	NS
MCP-1	1.2	NS
IL-6	1.1	NS
IL-10	1.1	NS
CINC-2 (MIP-2)	2.4	0.01
IFN-γ	0.5	0.045
Fractalkine	0.2	0.02
GM-CSF	0.4	0.048
LIX	0.9	NS

Values are expression relative to levels in cells cultured in glucose (5.6 mM) with 100 μM L-cysteine but lacking homocysteine; n = 3. *Abbreviations*: TIMP-1 - tissue inhibitor of metalloproteinases 1; TNFα- tumor necrosis factor alpha; β-NGF - *beta *nerve growth factor, MIP-3α- macrophage inflammatory protein 3 alpha; MCP-1 - monocyte chemotactic protein 1; IL-6 - interleukin 6; IL-10 - interleukin 10; CINC-2 - cytokine-induced neutrophil chemoattractant 2 (also known as macrophage inflammatory protein 2 and GROβ [growth-regulated gene product beta]); IFN-γ - interferon beta; GM-CSF - granulocyte monocyte colony stimulating factor; LIX - Lipopolysaccharide (LPS)-induced chemokine.

### Homocysteine induces MIP-2 expression and increases MIP-2 protein

**Figure 1:**
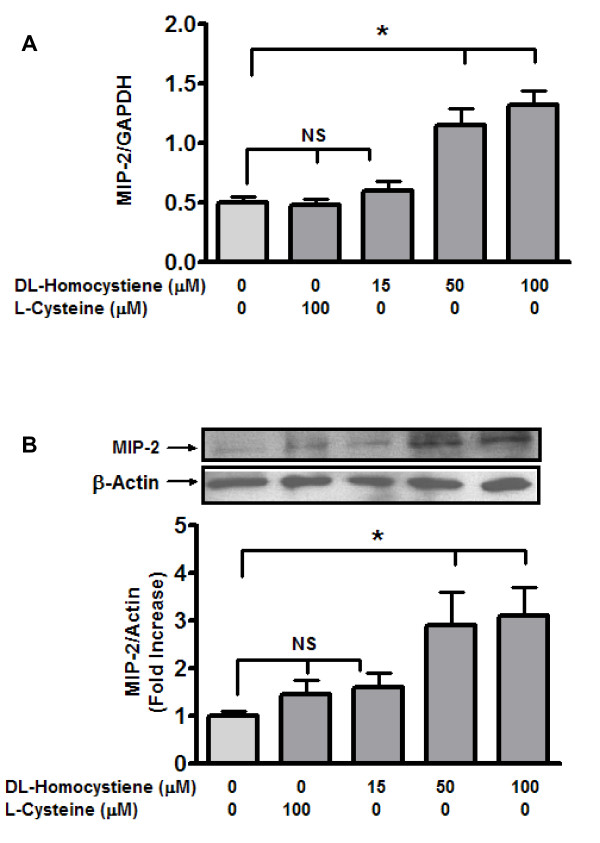
**Homocysteine induces MIP- 2 mRNA (A) and Protein (B) in mesangial cells**. MCs were incubated with L-cysteine (100 μM) or Hcy (15 μM, 50 μM and 100 μM) for 24 hours at 37°C in 100 mm dish. To determine expression (**A**), following trypsinization of cell monolayers, total RNA was isolated by the single-step method [19]. Subsequently, qRT-PCR was performed as described in text. Total protein was extracted from harvested cells under non-denaturing conditions using lysis buffer. MIP-2 protein levels (**B**) were detected by western blot. Results are representative of three separate experiments. Protein bands were quantified (Quantity One software, Bio-rad) and levels were represented as percentage response of control (100 μM L-Cysteine). Data represent mean ± SEM from three separate experiments. *p < 0.05.

**Figure 2:**
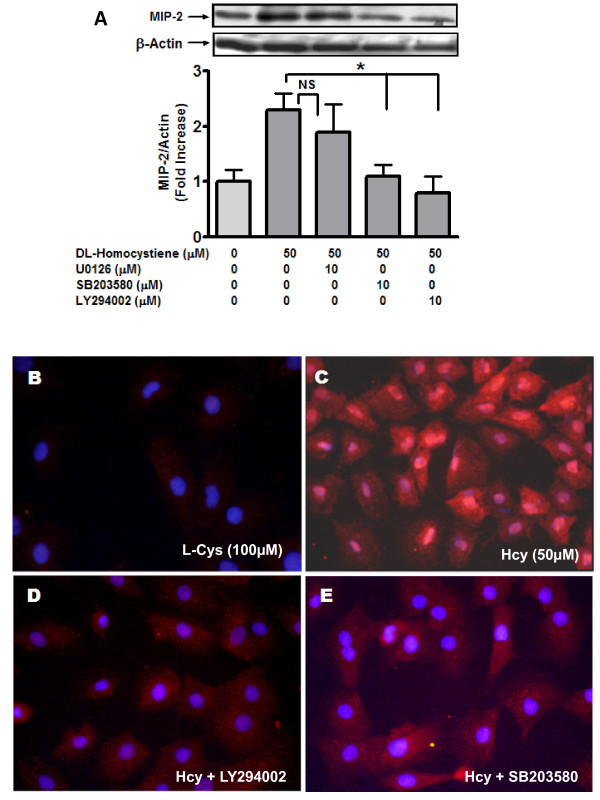
**Homocysteine-induced MIP- 2 is mediated by p38MAPK and PI3 kinase**. MCs were incubated (24 hours; 37°C) in the presence of Hcy (50 μM) with or without inhibitors U0126 (p42/44 MAPK inhibitor; 10 μM), SB203580 (p38MAPK inhibitor; 10 μM) and LY294002 (PI3 Kinase inhibitor; 10 μM). Cells were washed with PBS (4°C) and harvested using lysis buffer under non-denaturing conditions. MIP-2 protein was detected by western blot (**A**). Subsequently, protein bands were quantified as before. Results are representative of three separate experiments. Data represent mean ± SEM; *p < 0.05 indicate significant inhibition compared to 50 μM Hcy. (**B to E**) MCs were incubated (24 hours; 37°C) in the presence of Hcy (50 μM) with or without kinase inhibitors in Lab-Tek II dual chamber slides (Nalge Nunc, Naperville, IL, USA). The fixed MCs were immuno-stained with rabbit polyclonal GRO beta antibody followed by Alexa-Fluor 555 conjugated anti-rabbit antibody as described in the method. Nuclei were stained with DAPI. Panel B: L-Cys [100 μM], Panel C: Hcy [50 μM]; Panel D: Hcy [50 μM] + LY294002 [10 μM]; Panel E: Hcy [50 μM] + SB203580 [10 μM]. Panels are representative of 3 separate experiments.

Initially we determined the influence of variable Hcy concentrations (15, 50 and 100 μM) on MIP-2 expression by qRT-PCR. The results (figure 1A) indicated a significant impact on expression at 50 and 100 μM. Another sulphur-containing amino acid (L-cysteine), that is structurally similar to DL-Hcy [24] did not influence expression. Hence changes in MIP-2 expression can be attributed to an effect specific to Hcy, rather than to structural similarities with L-Cys. Subsequently, the expression of MIP-2 induced by Hcy in MC was quantified by western blot analysis. In line with the expression data, Hcy significantly increased MIP-2 protein levels in MC (figure 2B). Of note, MIP-2 expression increased 2.5 fold at 50 μMHcy, compared to expression at 100 μM L-Cys (p < 0.05). MIP-2 levels did not increase further when Hcy concentration was increased to 100 μM.

### Homocysteine induced MIP- 2 requires p38MAPK and PI3kinase but not P42/44 MAPK Signaling

MIP-2 induction has been reported to be MAPK and PI-3 Kinase dependent [25]. Hence, we investigated role of MAPK and PI-3 Kinase in MIP-2 expression induced by Hcy. Hcy-induced MIP-2 was significantly attenuated (p < 0.05) by a PI-3 Kinase inhibitor (LY294002) and by an inhibitor of a p38MAPK (SB203580). In contrast, use of a p42/44 MAPK inhibitor (U0126) did not significantly alter Hcy-induced MIP-2 (figure 2A).

Immunohistochemistry was employed as another analytical tool to examine the effect of Hcy on mesangial MIP-2. Cells were exposed to Hcy (50 μM/0.5% FBS), in the absence and presence of inhibitors to p38MAPK (SB203580; 10 μM) and PI3 Kinase (LY294002; 10 μM). MIP-2 expression in medium supplemented with FBS (0.5%) and L-Cys (100 μM) represented control conditions. As revealed in figure 2, panel C, the expression of MIP-2 was increased by Hcy (50 μM) compared to control (panel B). Hcy-induced of MIP-2 was abolished by LY294002 (PI3 Kinase inhibitor; panel D) and SB203580 (p38MAPK inhibitor; panel E). These results suggest that Hcy induced expression of MIP-2 in MC was mediated by p38MAPK and PI-3 K signalling pathways and are consistent with the results derived from Western blotting analysis.

### Hcy activates p85 PI-3 Kinase and p38MAPK in mesangial cells

In an effort to corroborate the observations related to blunting of the effect of Hcy on MIP-2 by inhibitors of PI3 Kinase and p38MAPK, western blotting analyses was employed to determine levels of activated (phosphorylated) p38MAPK and PI3 Kinase in MC exposed to elevated levels of extracellular Hcy.

**Figure 3:**
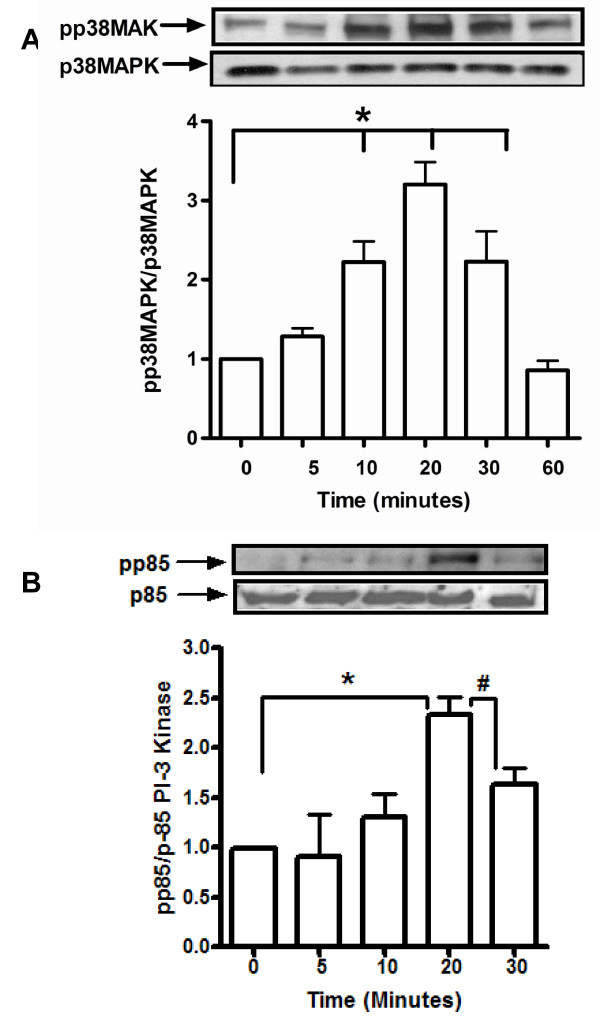
**Hcy increases phosphorylation of p38MAPK (A) and p85 PI3 kinase (B)**. Mesangial cells were serum-starved overnight prior to exposure to medium containing L-cysteine (100 μM) or Hcy (15 μM, 50 μM and 100 μM). Cells were washed with PBS (4°C) and harvested using lysis buffer under non-denaturing conditions. Total p38 MAPK, total p85 PI-3K, phosphorylated p38 MAPK and phosphorylated p85 PI-3 Kinase expression was detected by western blot as described in methods. Protein bands were quantified and the ratios of pp38MAPK/p38MAPK and pp85/p85 were represented as fold-changes compared to t=0. Panel depict representative blot of three separate experiments performed in duplicates; values are expressed as mean ± SEM; *p < 0.02; #p < 0.05.

Hcy induced time dependent increases in p38 MAPK phosphorylation between 10 and 30 minutes. Phosphorylation of p38 MAPK decreased significantly at 60 minutes as compared to that for 10 minutes (figure 3A). Similarly, Hcy induced p85 PI3K phosphorylation in a time dependent manner. Phosphorylation of p85 PI-3K significantly increased at 20 minutes (2.25 fold as compared with levels at the initiation of the study). At 30 minutes, p85 PI-3K phosphorylation decreased as compared with 20 minutes (figure 3B).

### MIP-2 Modulates Leukocyte cell adhesion to mesangial cells

**Figure 4:**
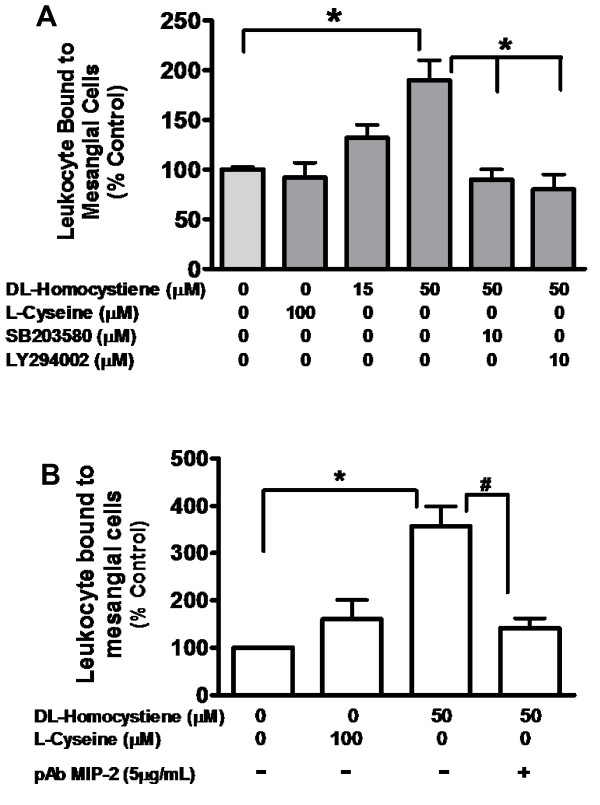
**Hcy-induced leukocyte cell adhesion to mesangial cells is abrogated by p38MAPK and PI-3 Kinase inhibitors (A) and by anti-MIP2 antibody (B)**. MC were incubated (24 hours/37°C) in presence of Hcy (50 μM) with or without inhibitors SB203580 (p38MAPK inhibitor; 10 μM) or LY294002 (PI3 Kinase inhibitor; 10 μM) or in the presence of pAb MIP-2 (5 μg/ml) B. L-Cys (100 μM) was used as a control. Cell adhesion assay was performed as described in method. The data represent mean ± SEM from three separate experiments; *p < 0.05; ^#^p < 0.02.

Hcy-induced leukocyte adhesion to MC was determined by cell adhesion assay following incubation of with Hcy; L-Cys (100 μM) represented control condition. L-Cys (100 μM) did not have a significant effect on leukocyte adhesion to MC whereas Hcy induced dose dependent increase in leukocyte adhesion to mesangial cells. Leukocyte adhesion increased significantly up to 1.8 fold (P < 0.02) at 50 μM Hcy compared with control condition (figure 4A).

SB203580 and LY294002 treated MC was employed to determine the role of p38MAPK and PI-3K in MIP-2 mediated leukocyte adhesion to these glomerular cells. As revealed (figure 4A), LY294002 (PI-3 kinase inhibitor) and SB203580 (p38MAPK inhibitor) blocked leukocyte adhesion induced by 50 μM Hcy (P < 0.05). Blocking antibody against MIP-2 (5 μg/ml) confirmed the functional role of MIP-2 in Hcy-induced leukocyte adhesion to MC. Hcy (50 μM) induced leukocyte adhesion to MC was significantly blocked up to 3 fold by MIP-2 antibody (p < 0.01) (figure 4B).

## Discussion

MIP-2 is a C-X-C chemokine, known to recruit neutrophils [26] and studies suggest that neutrophil recruitment may bear relevance to the development and progression of glomerular diseases. The initial indication that MIP-2 may participate in glomerular disease arose from observations that isolated glomeruli and MC produced MIP-2 in response to immune complexes [27]. Subsequently, in another *in vivo *rat model of mesangioproliferative glomerulonephritis (MPGN), glomerular nitric oxide (NO) was shown to be capable of inducing MIP-2 expression, which in turn lead to neutrophil recruitment [12]. Kidney disease is associated with increases in plasma Hcy [28] and Hcy induces MCP-1 production by glomerular MC [8]. In order to identify cytokines whose expression may be increased by Hcy, we initially employed antibody array approach to evaluate cytokine production by MC exposed to pathophysiologic levels of Hcy.

Our initial observation (table 1) was that elevated extracellular Hcy increased the levels of cytokines, TIMP-1 (1.9-fold) and MIP-2 (2.4-fold). For another cytokine, MCP-1 there was a 20 percent increase in protein levels, but this was not statistically significant. Other studies have demonstrated a 20 to 40 percent increase in MCP-1 by MC [8] and hepatocytes [29] exposed to comparable concentrations of Hcy. Hence, our observations are similar to the aforementioned reports, but in the current study, Hcy-induced MCP-1 changes were not significant. In contrast, the observations for TIMP-1 are consistent with earlier studies [30,31], while data relating to induction of MIP-2 by Hcy have not been previously reported. Accordingly, we explored the influence of Hcy on MIP-2 expression in MC and examined potential signalling mechanism(s) that may mediate this process.

In support of the antibody array data (table 1), we observed that in MC exposed to Hcy there was a significant increase in MIP-2 expression and protein with changes occurring at Hcy concentrations of 50 μM and 100 μM respectively. These observations are in line with those that have been reported for other cellular processes that are affected Hcy [9,10]. Subsequently, we chose to examine downstream signaling that may be involved in this effect of Hcy on MIP-2 expression in MC. In an earlier report, hypoxia-induced MIP-2 expression in macrophages was shown to be dependent on p42/44 MAPK and PI-3 kinase pathways [25]. In another study, TNF-α induced MIP-2 in cultured mouse astrocytes was mediated via both p42/44 MAPK and p38 MAPK [32]. Accordingly, we studied the impact of inhibitors of p42/44 MAPK, p38 MAPK and PI3 Kinase on Hcy-induced MIP-2 in MC. Indeed, we observed that Hcy-induced MIP-2 expression was inhibited by PI-3 kinase inhibitor (LY294002) and p38MAPK inhibitor (SB203580), but was unaffected by p42/44 MAPK inhibitor (U0126) (figure 2). Thus, our observations are consistent with earlier reports demonstrating that MIP-2 is regulated by specific kinases [33,34]. The failure to demonstrate a role for p42/44 MAPK signalling in Hcy-induced MIP-2 in the current study may be related to the type of cells be studied.

Our earlier study revealed that Hcy activates p38MAPK [9]. Accordingly, we examined the effect of Hcy on phosphorylation of p38MAPK and p85 (catalytic subunit of PI3 Kinase). As revealed in figure 3, Hcy induced time-dependent increases in phosphorylated species of p38 MAPK and p85 subunit of PI3 Kinase in MC. Vascular smooth muscle cells (phenotypically related to MC) manifest MAPK- and PI3-K-dependent increases in MMP-2 synthesis upon exposure to Hcy [35]. Other studies have identified a role for MAPK activation in mediating MIP-2 production by renal tubules and peritoneal macrophages [33,34]. Although the stimuli and cell type are different, the observations in the current study relating to Hcy-induced p38MAPK and PI3 Kinase activation are consistent with those reported in other studies.

Leukocyte infiltration and subsequent interstitial inflammation are emerging as key features of various glomerular diseases [11,36]. These observations have been validated in various modular systems [37-39]. In order to determine potential consequence(s) of changes in Hcy-induced MIP-2 expression, we studied leukocyte adhesion to MC using an *in vitro *protocol. 'In this regard, the initial observation was that Hcy increased leukocyte binding to MC (p < 0.05) while L-Cys was without effect (figure 4A). Furthermore, inhibition of p38MAPK and PI3K activation abrogated Hcy-induced leukocyte bound to MC (figure 4A). Finally, we were able to validate that MIP-2 mediated leukocyte adhesion to MC by demonstrating that polyclonal MIP-2 antibody (5 μg/ml) was capable of blocking leukocyte adhesion to MC pre-incubated with Hcy (50 μM).

## Conclusion

The current study reveals that Hcy induces MIP-2 expression in MC and that this effect is dependent on both PI-3 Kinase and p38MAPK activation. Furthermore, MIP-2 may be important in PI-3 Kinase- and p38MAPK-dependent leukocyte adhesion to MC. The results highlight a link between MC production of MIP-2 and its potential role in leukocyte adhesion to MC. This is pertinent to kidney disease because elevated plasma Hcy is a hallmark of progressive kidney disease and endstage kidney failure. Future *in vitro *and *in vivo *studies are required to further ascertain the consequences of Hcy-induced MIP-2 expression in glomerular MC.

## List of Abbreviations

CKD: chronic kidney disease; Cys: cysteine; ESRD: endstage kidney disease; DMEM: Dulbecco's Modified Eagle's Medium; ESRD: Endstage Renal Disease; FBS: fetal bovine serum; GFR: glomerular filtration rate; Hcy: homocysteine; Hhcy: hyperhomocysteinemia; MCP-1: marcophage chemoattractant protein 1; MC: mesangial cells; MAPK: mitogen activated protein kinase; NF-κB: nuclear factor kappa B; PI3 Kinase: phosphatidyl inositol 3-Kinase; PBS: phosphate buffered saline; SDS - PAGE: sodium dodecyl sulphate -polyacrylamide gel electrophoresis; TBS: Tris buffered saline; TTBS: Tween-Tris buffered saline; TIMP-1: Tissue inhibitor of metalloproteinase 1.

## Competing interests

The authors declare that they have no competing interests.

## Authors' contributions

SS and LRJ conceived of and designed the studies. The experimental work, data collection and interpretation and, as well, manuscript preparation were performed by SS and LRJ.
